# Arsenious Acid in Dentistry

**Published:** 1878-12

**Authors:** J. Hardman

**Affiliations:** Muscatine, Iowa


### ARSENIOUS ACID IN DENTISTRY.

BY J. HARDMAN, D. D. S., MUSCATINE, IOWA.

*Read before the Iowa State Dental Association.*

*Mr. President and gentlemen of the Iowa Dental Association:* The white oxide of arsenic, as a drug, is sufficiently familiar to those who may constitute my hearers to preclude an attempt on my part to present its pharmacy. It is with its nature of medicinal character and uses wherewith we have principally to deal, and in which we are almost solely interested.

This agent was introduced into dental therapeutics about forty years ago by Dr. Jno. R. Spooner, then of Montreal. And to him no small debt of gratitude is due from the dental profession on the one hand and the victim of diseased teeth, as patient, on the other. It must, indeed, have been a hero that dared to step forward, isolated and alone, with so potent a weapon in hand as the accredited arch poison of almost all toxicologists, But he proved himself fully able to the task, and demonstrated in every attack made upon its claims the rectitude of this theory and thus stamped his name with a lasting immortality.

We find a wide range of opinion amongst authors as to its proper place in the materia medica. Dunglison, Eberle and others pronounce it "tonic and escharotic." Wood, Taylor, Christison and others equally eminent say, "it is not escharotic nor corrosive." Wood asserts "it does not act chemically, but is virulent through its influence upon vitality in the organism." Prof. J. F. Flagg, in the *Dental Cosmos* of '68, has given us probably the most comprehensive and rational theory of this subject in these brief words, "It is a vital irritant."

One thing is certain, that the practice with this drug has been as vague and diversified as its accredited medical status; and like many others, it has been very empirically used. And could we believe its various advocates, it has cured almost every ailment man has been heir to.

That to be the better qualified to use this potent agent successfully, and to the greatest extent of usefulness we should at least well understand its *modus operandi*, so far as it may affect our practice with it in dentistry. Bearing, then, in mind that this drug, like many others, is modified in its effect by various causes, as may be instanced, in general or local exhibition; large or small in dose; physiological or pathological condition of organism; whether on living or dead tissue; whether on tissue of high or low vitality, etc., etc. We offer the following definitions which we think may be accepted as safe in practice:

1. That arsenious acid is not escharotic or corrosive.
2. It does not act chemically, strictly speaking.
3. It is not an antiseptic on vital tissue.
4. It is an antiseptic when used on dead organized tissue.
5. It is an irritant on live tissue through the influence of vitality; and by this combined capacity becomes the powerful agent and virulent poison that it is.

The seeming paradox that, although the agent is indeed a powerful antiseptic on dead tissue, yet probably never preserving the devitalized pulp from disorganization and sloughing, is, we think, comprehensible enough when we remember that it has been satisfactorily demonstrated that not more than the one-millionth of a grain is absorbed into the structure of the dead pulp. (See Prof. J. Foster Flagg in *D. Cosmos* of 1868,) This quantity being too insignificant to establish a preserving influence; but yet amply large enough to destroy the life of this very vital organ. That this minute quantity can accomplish so much is made manifest when we consider the dimensions of the apex foramen—the passage is readily and completely impeded by the irritation and its consequences caused by this agent.

Then we observe that arsenious acid is no antiseptic on vital tissue; yet nevertheless a powerful one on organic dead tissue when used in sufficient quantity. An infinitesimally small quantity will act as an irritant on vital tissue and the higher the vital organism the more virulent is its action.

Neither can the constant sloughing of the pulp and its appendages be caused by the direct presence of this agent in its structure, but a result which would be the same if the animation had been cut off as suddenly and completely by any other agent. Hence the speedy disintegration that takes place on the death of the pulp is not in the least arrested by the presence of this agent in its structure as the amount is not an antiseptic quantity.

Furthermore, it is found that, although modified action is observable when congestion and even inflammation of the pulp exists prior to, and at the time of the application, yet the result is so very similar that we need not dwell to trace in detail the facts.

In order the more clearly to bring this subject before us we shall announce these two general divisions:

First—The use of arsenious acid in dental practice.

Second—Its toxical character and the needs of caution by the practitioner.

Under this first division it would be natural to note what has been done in practice by this agent, and by whom. Whether as an empiricism merely by quacks, or whether by the honorable and highly qualified in the profession by prescription at once scientific and comprehensible. We confess that the facts are that nearly all in our profession have handled this agent very similarly. That but little short of empiricism or mere routine has been the general habit and rule; and that the few who have well considered its proper ties and effects, the exception. This fact impresses us with the manifest needs existing that we better study and understand this agent, and thus the better know how and when and where to use it.

To those who condemn the practice of devitalization of the pulp in toto, we have but this to say, although to argue this point pro and con is entirely beyond the reach of this paper. We think the cause of humanity itself justifies it, and that nothing has contributed to its condemnation so much as abuse and malpractice.

Arsenic has from its first introduction into our practice been almost exclusively used for devitalizing the denital pulp. Whatever may, or may not have been the prevailing opinion as to the propriety or rectitude in ever devitalizing this organ, the practice still prevails, and this same agent holds the first rank.

The first to combine creosote with it for this purpose was Dr. Flagg, Sen., of Philadelphia, in 1863. This adjunct formed an excellent compound, both in convenience of application, and in medical efficiency, and a paste of simply arsenious acid and creosote is still very generally the favorite form. Although some prefer the further addition of morphine or other narcotic. We are of the opinion, however, that these add nothing of advantage to the simple paste.

This paste is usually worked into a minute pledget of cotton wool, and after removing the disintegrated material from off of the pulp as much as is bearable, it is placed neatly over and in contact with the pulp, and over this is applied another and sufficiently large pledget of cotton wool saturated with an alcoholic solution of sandarac, shellac, mastic, or some other waterproof gum, and left to remain from one to two days. At the end of a proper extent of time the pulp will generally be found, and in most cases sloughing—in a semi fluid state, and can be removed without causing pain.

It has been a prevailing habit of authors of our text books to caution against leaving the application in the cavity over twenty-four hours, This we think often leads to imperfect subsequent treatment, as owing to an attempt to remove the pulp and its appendages before the sloughing stage, may result in leaving behind portions that will eventually contribute to trouble. We therefore advise that a period of at least two days, and in most cases a week or fortnight is best. And here we find we are again supported by that indomnitable investigator, Prof. Flagg, in the *Dental Cosmos* of 1868, in these words, "I therefore instituted a gradual increase of duration of applications, watching carefully the cases, until year after year told me that I could with perfect safety permit them to remain until the pulps were dead and had sloughed."

Again, we have been taught, (and the sentiment has generally prevailed), by our instructors that the application of arsenic is not correct where either pulpitis or peridonitis prevails. We see no good reason for this, and our experience has so confirmed us that there is no need of preparative treatment. No matter what the amount of pain or inflammation, if the tooth is worth retaining, the pulp vital, but deemed best to sacrifice the same, then apply the arsenical application at once, and, if properly done, the best results will follow. Indeed, in severe odontalgia from pulpitis there is not to my knowledge so efficient and certain a remedy as this.

There is occasionally a soreness in the connecting membranes of the tooth that has been under arsenical treatment, that has been imputed to the result of an absorption of the drug into these structures, through the apex foramen. This view we think incorrect as a rule. That there are cases where this agent may pass the apex foramen in quantity to do violence is possible, and we propose to consider the same as we proceed farther, but wish it here to be understood, that we hold, that as a general rule this soreness is only the result of the death of the dentinal nerves and vessels, and the consequent irritation, and the conditions might and would be the same if destroyed by any other agent. This soreness usually requires but little attention. Usually subsiding in a short time without special treatment.

The next, and what we regard as the most important use this agent may be made to subserve in the dental practitioner's hands, is that of denuding sensitive dentine. We are aware that here we are advancing upon disputed territory, and may seemingly appear to again bring to the front a controversy that in years past raged warm, and was by the survivors declared settled against, and in condemnation of this practice. Let that be as it may, and although the theory is not to be eschewed, yet we think that knowledge gained and backed by experience is most trustworthy and reliable in practice. And here we know whereof we speak. Feeling confident that it surpasses all other agents ever brought to our notice for this purpose in efficiency, and, at the same time perfectly safe in regard to the security of the vitality of the tooth. Of course we refer only to those cases of extreme sensitiveness of dentine where the pulp is not exposed, but where there is still remaining some dentine covering.

It is very evident to us that this agent has no effect, chemically or otherwise, to injure the dentine itself. Neither do we entertain the least idea, as some have done, that it may enter the dentinal tubuli, and thus finally effect deleteriously the pulp. We have had frequent opportunities to observe the unimpaired vitality of teeth, from five to fifteen years after being treated for sensitive dentine, being treated for sensitive dentine, where the application of arsenic had remained in the tooth for the period of forty-eight hours, and never have we seen a single case wherein this agent had evidently destroyed the life of the pulp, where the said pulp still had some healthy dentine covering it at the time of application. And we have used it in many cases for this purpose, and on even quite young subjects. You will bear in mind that this agent is an irritant only when in contact with vitality, and the degree of its virulency is in proportion to the grade of said vitality. Then, where there is but inferior vitality, as in tooth structure, the consequent irritation, if any, must be comparatively slight, even upon the surface of dentine where it comes into positive contact. There is then not the remotest danger offered the pulp or any other very vital part of the tooth.

We use it for this purpose in the same manner of application as for the devitalization of the pulp, except as to duration of time. From six to twenty-four hours is generally sufficient. Yet a much longer time will do no harm. The application should remain in situ up to the time of excavation, or the sensitiveness again, in some cases partially returns.

But one other use for arsenious acid in dentistry we would mention. (I think the credit for first using arsenious acid for this purpose is due Dr. J. D. White, of Philadelphia). That is, its power to render more practicable the removal of teeth that are sometimes found so obstinately attached in their sockets, as to preclude their safe removal by a mere immediate force with the forceps.

I will illustrate this by giving a case. Mr. N———, aged about forty, of sanguino-bilious temperament, wished the removal of an only remaining canine tooth. We placed a well selected forceps upon it and made traction, the patient grasping with both of his hands and added to ours his entire strength, yet not in the least affecting our object. We removed the instrumeut, and being told by the patient that he felt symptoms of the maxilla giving away just below the eye, we concluded best to desist.

We then, with a thread saturated with arsenical paste, ligated this tooth, pushing the thread well up under the gums, and then passed several turns of dry thread over it, which we well saturated immediately with sanderac solution. Left all remain for forty-eight hours, at which time, after removing dressings, we found the gums some tumid and quite black around the neck of the tooth; the tooth sore and some loose. We applied the forceps once more and readily removed the tooth, with, we should judge, not using one-third the tractive force used upon it two days previous. The edges of the socket seemed somewhat divested of periosteum, but a speedy healing followed as usually occurs in ordinary cases. We have repeated this practice in several instances and with invariable success.

It now remains for us to consider before closing, the character of this agent in reference to some of its topical tendencies when used in the practice of dentistry. If one case is negiected dire misfortune may follow as a sequel. Hence, we can not feel justified to leave unwarned the party whose duty calls him to use this potent agent upon his confiding patrons.

The circumstances under which injury may be the more likely to occur are:

First—Imperfect and bungling mode of application; and

Second—In over treatment in devitalizing pulps, especially in young subjects.

First—We have seen a few cases of injury caused by inattention and want of care while making the application. And in one of these I "had a finger in the pie." Especially is there cause for care when it is intended to make application to devitalize where the cavity extends under the edge of the gums. In cases where injury has thus been the result of carelessness these may be the sequel: On the following day the gums in the festoons between the teeth will be swollen and dark; tooth some sore on percussion. Third, fourth and fifth days symptoms much the same, save that some alteration is observable, and on probing the sensibility and periosteum is found gone from the alveolus between the teeth where the application was made. On sixth or seventh day dark color of gums gone, more ulceration, and the now bare alveolar process between the teeth is, on grasping, found loose, which, when removed, leaves a wound of greater or less extent, and, although usually readily heals, and yet the permanent loss of so much osseous tissue ever remains to tell of the botchy practice that may thus follow to distress the patient and mortify the operator. This narrates not the greatest consequences of injury that may arise from such acts of carelessness, but some of you have very likely seen far worse cases.

Secondly—There is danger of over treatment, and this may occur in more than one way. An application may be removed by other parties than that of the one who made it. An interval of inattention by the patient of weeks may supervene, when pain from peridontitis starts him to some other dentist who may, on the request of the patient, try his hand at "killing the nerve," and, without a clear diagnosis or history of the case, applies the agent to an empty chamber that may communicate to the socket by means of the apex foramen and through this avenue it passed into the deep tissues of the jaw, and the results may be truly horrible. Irritation, followed by ulceration of both soft and hard tissues of the maxlliary, with permanent deformity, untold suffering and, may be, death.

Permit the relation of a single case, (and not the worst we have seen), for illustration:

Mr. ———, a traveling clerk of a Chicago house, aged about thirty-five and of good constitution, called at our office about five o'clock p. m., with a request that we "put a little arsenic in" his tooth, with lateral cavity extending into the pulp chamber, which was empty. Tooth sore, gums red and swollen, and tooth seeming to be swimming in pus.

We said "No, this tooth can have no arsenic treatment, but the forceps is the proper remedy now." Patient not being prepared for this summary announcement, refused our prescription, and promised to call in the morning.

This was the history of the case as he gave it: "Had arsenic put into the tooth just before leaving Chicago. Next day at La Salle had tooth cleaned out and had arsenic again put in. Second day after, (which was yesterday), was at Davenport and had arsenic again put in. I thought as it was still hurting me it needed more arsenic, and I called for that purpose."

In the morning he called as he had promised to, and as he stood in the doorway he held up between thumb and finger the tooth, saying, "That's him, I picked him out easily with my fingers." The root was as free from peridentium as though it had been scraped off on purpose. We invited him into the chair, and on inspection found a point of bear alveolus protruding which we grasped with small tweezers and brought away readily the entire socket, forming a perfect sheath for said tooth.

In this case is furnished material for an extended communication itself. We will but remark, however, that it is our firm conviction that had there but one application been made, and that allowed to remain even double the entire time, no untoward results would have presented. The mischief came in the reapplications into an empty pulp chamber and root canal and allowing the drug to pass through the apex foramen, and thus establishing the specific effect of the agent on the vital tissues in the jaw around the tooth.

Again, special cause for caution is found in the treatment of immature teeth in young snbjects. In such cases the grade of vitality is active, and through this a greater liability to extensive injury is presented. Then another reason of danger in such cases is, that the apex foramen is very much larger in such teeth, and this may favor a ready transit for the drug. Cases might be cited showing results arising for want of due caution in such cases that have caused the direst regrets. But this communication is becoming too lengthy and we will desist, adding that in cases of young persons where the teeth are maturing, and it is resolved to devitalize the application had best remain but twenty-four hours, and its removal not entrusted to the patient, as you will many of you bear me witness that the patient for want of proper information is often found to assume the prerogative of knowing best how long it should remain, and how often reapplied "to guarantee the sure death of the aching nerve." And should the patient have attempted the removal, will have most probably taken away but the covering and left behind the acid, uninterrupted in the cavity.

In closing permit me to appeal, to especially the younger practioner, that while ample opportunity exists in which this agent can be made to contribute largely and safely to the accomplishment of much usefulness, yet a vigilant and intelligent discrimination is incumbent upon him morally, honorably and professionally, to avoid the snares and dangers your humble servant has feebly, though earnestly attempted to portray.

